# Size-controllable synthesis and bandgap modulation of single-layered RF-sputtered bismuth nanoparticles

**DOI:** 10.1186/1556-276X-9-249

**Published:** 2014-05-21

**Authors:** Bin-Kun Wu, Ming-Yau Chern, Hsin-Yen Lee

**Affiliations:** 1 Institute of Biomedical Engineering, National Taiwan University, Taipei 10617, Taiwan; 2 Department of Physics, National Taiwan University, No. 1, Sec. 4, Roosevelt Rd, Da'an District, Taipei 10617, Taiwan

**Keywords:** Bismuth, Nanoparticle, RF sputtering

## Abstract

We here report a simple and efficient method to grow single-layer bismuth nanoparticles (BiNPs) with various sizes on glass substrates. Optimal conditions were found to be 200°C and 0.12 W/cm^2^ at a growth rate of 6 Å/s, with the deposition time around 40 s. Scanning electron microscope (SEM) images were used to calculate the particle size distribution statistics, and high-resolution X-ray diffraction (XRD) patterns were used to examine the chemical interactions between BiNPs and the substrates. By measuring the transmission spectra within the range of 300 to 1,000 nm, we found that the optical bandgap can be modulated from 0.45 to 2.63 eV by controlling the size of these BiNPs. These interesting discoveries offer an insight to explore the dynamic nature of nanoparticles.

## Background

Bismuth (Bi) is a group V semi-metallic element with a rhombohedral crystal structure commonly indexed to a hexagonal lattice (*a* = 4.574 Å, *c* = 11.80 Å). Several interesting physical properties, such as highly anisotropic Fermi surface, low carrier density, small carrier effective mass, large Fermi wavelength (40 ~ 70 nm at room temperature), and long mean free path (100 nm at room temperature and 400 μm at 4.2 K) [1-5], have made it a potential candidate for many interesting applications. For example, electrodes incorporated with Bi nanostructures can be used to detect heavy metals (such as Pb^2+^, Cu^2+^, Zn^2+^ and Cd^2+^) in water solution, replacing the traditionally toxic mercury materials [6-8]. Moreover, some of the Bi binary compounds, such as bismuth telluride (Bi_2_Te_3_) and bismuth selenide (Bi_2_Se_3_), are efficient thermoelectric materials [9,10], and interesting effects related to the temperature dependences of the Seebeck coefficient can be found in Bi nanowires (BiNWs) [11,12]. More recently, these Bi compounds were used in the first experimentally realized three-dimensional topological insulator state in bulk solids [13,14]. Bi nanoparticles (BiNPs) also have been specifically useful in biological science, such as bioimaging [15] and biosensing [16]. As far as preparation of high-quality BiNP samples is concerned, the main challenges remain on the size and morphology control and the lack of sufficient understanding to achieve this control, since the electrical, magnetic, and optical properties of metal nanoparticles depend strongly on the particle size and shape. The band structure of Bi also becomes size-dependent as the dimensions are reduced to the nanometer range, which can lead to a semimetal-semiconductor transition [17].

Generally speaking, BiNPs can be fabricated by several methods, including gas evaporation [18,19], simple chemical method [20-22], and e-beam evaporation [23]. Recently, other methods are also available [24,25]. All these methods have both advantages and drawbacks. For example, in the gas evaporation method, the mean particle diameter is controlled by molecular weight and pressure of the inert gas, which are convenient to produce various diameters of Bi particles. However, it is rather difficult to reproduce the same size with the same parameters. In the simple chemical method, BiNPs are prepared by using the thermal decomposition method of an aqueous precursor, for instance, Bi(SC_12_H_25_)_3_ or BiCl_3_. This method can prepare dense BiNPs in spherical shapes with enhanced thermoelectric properties, but the processing procedure is complicated, including the preparation of the self-made precursor. Also, it is almost impossible to fabricate BiNP arrays instead of particles that cannot be clearly identified. The e-beam evaporation method has the ability to grow BiNPs in a low deposition rate, but it is hard to control the uniformity of the evaporation rate due to the filament degradation in the electron gun.

Previously, we reported preparation of radio frequency (RF) sputtered BiNWs on glass substrates [26]. In order to study the growth mechanism, different growth temperatures, sputtering power densities, and deposition times were tested, and optimal conditions were found to be 120°C to 160°C, 0.5 W/cm^2^, and 240 s. The nanowires were straight and long (10 to 50 μm) with a well-defined square cross section. In this work, with suitable chosen parameters, the same experimental setup can be used to grow BiNPs. Compared to the growth of BiNWs, the deposition time and the power density to grow BiNPs are much lower. We were able to deposit BiNPs of various sizes by controlling the deposition time, as the diameters are directly proportional to the deposition time, and only a single layer of BiNPs are grown on the glass surface. Also, we further analyzed the sample quality and the absorption property in a statistical method.

## Methods

According to past experience, temperature is the most important factor to grow either a thin film, nanowires, or nanoparticles. Based on this, our strategy is to separate the experiment into three stages, which starts from searching for the best growth temperature. The first stage (experiment A) was to deposit Bi at several different temperatures, while keeping the power density and the deposition time fixed at 0.12 W/cm^2^ and 60 s, respectively. The second stage (experiment B) was to focus on the relationship between the particle diameter and the deposition duration. We deposited BiNPs with different deposition durations ranging from 10 to 60 s, with the deposition temperature maintained at 200°C and the power density at 0.12 W/cm^2^. The grain sizes of BiNPs were estimated by using a scanning electron microscope (SEM), and the bandgaps were determined by using the extrapolation method through measuring the visible-light absorption spectrum. The final stage (experiment C) was to deposit BiNPs on sapphire and ITO-coated glass (ITO glass) substrates. The reason why we choose these substrates as a part of our experiment is their possibility to fabricate linear or nonlinear optical devices for further applications. For example, different substrates can act as a light filter if we are interested in utilizing BiNPs to be convex lens for lasers.

We used Corning glass (Corning Inc., Corning, NY, USA) as our substrates in experiments A and B. Prior to deposition, all substrates (6 × 8 mm^2^) were ultrasonically degreased in acetone and alcohol for 10 min to remove contaminants, followed by rinsing in de-ionized water and drying under N_2_ flow. For all samples used in these three experiments, the argon pressure was maintained at 3 mTorr, the distance between the Bi target and substrate was 20 mm during growth, and a subsequent cool down process at a rate of −8°C/min brings the sample back to room temperature.

The surface morphology was examined by a LEO 1530 field emission SEM (LEO Elektronenmikroskopie GmbH, Oberkochen, Germany). Structural characteristics were measured by using the high-resolution X-ray diffraction (XRD) method with a Bede D3 diffraction system and a Mac Science M21X X-ray generator (MAC Science Co., Ltd., Yokohama, Japan). High-resolution measurement was performed by using a parabolic Göbel mirror and a pair of channel-cut monochromating crystals in (+, −) setting, where the total divergence of the Cu Kα_1_ line is less than 10 arc sec. Optical transmittance was measured by a monochromatic Xe lamp and an Acton Research Corporation SpectraDrive spectrometer (Acton Research Corporation, Acton, MA, USA), and the incident light power data acquisition was recorded by a Newport dual-channel power meter model 2832-C power meter (Newport Corporation, Irvine, CA, USA). The parameters of each sample in the experiment are listed in Tables 1 and 2.

**Table 1:** **List of BiNPs samples grown at 0.12 W/cm**^**2 **^**with different deposition temperatures and time**

**Number**	***T *** **(°C)**	***P *** **(W/cm**^**2**^**)**	***t *** **(s)**	**Number**	***T *** **(°C)**	***P *** **(W/cm**^**2**^**)**	***t *** **(s)**
Bi-101	RT	0.12	60	Bi-201	200	0.12	10
Bi-102	60	0.12	60	Bi-202	200	0.12	20
Bi-103	100	0.12	60	Bi-203	200	0.12	30
Bi-104	160	0.12	60	Bi-204	200	0.12	40
Bi-105	200	0.12	60	Bi-205	200	0.12	50
Bi-106	240	0.12	60	Bi-206	200	0.12	60

**Table 2:** **List of BiNP samples grown at 0.12 W/cm**^**2 **^**with different deposition temperatures**

**Number**	**Substrate**	***T *** **(°C)**	***P *** **(W/cm**^**2**^**)**	***t *** **(s)**
Bi-301	ITO glass	160	0.12	60
Bi-302	ITO glass	200	0.12	60
Bi-303	c-Al_2_O_3_	160	0.12	60
Bi-304	c-Al_2_O_3_	200	0.12	60

## Results and discussion

**Figure 1:**
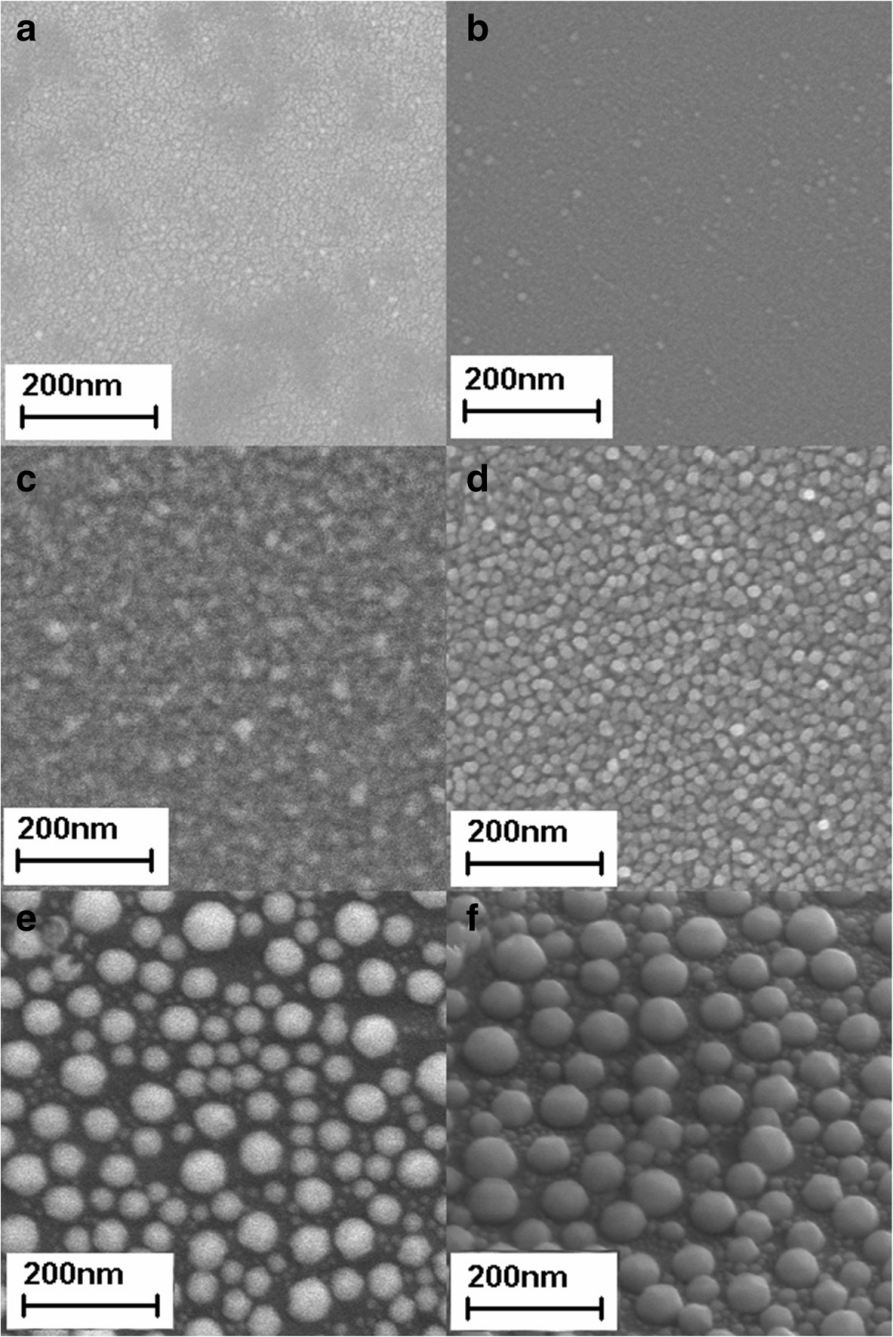
**SEM images of BiNPs deposited on glass substrates at different temperatures. (a)** RT, **(b)** 60°C, **(c)** 100°C, **(d)** 160°C, **(e)** 200°C, and **(f)** 240°C, which correspond to sample Bi-101 to Bi-106 in Table 1.

The SEM images of BiNPs of experiment A at six different temperatures (RT, 60°C, 100°C, 160°C, 200°C, and 240°C) are shown in Figure 1. Samples grown at low temperatures (RT, 60°C, and 100°C) can only be regarded as Bi thin film samples. These samples have smooth surfaces with only a small amount of tiny BiNPs. Samples grown at high temperatures (160°C, 200°C, and 240°C), however, have a large amount of BiNPs. This observation can be clearly understood: in a low-temperature environment, the sputtered Bi composites do not have enough time to form larger crystals before being frozen. At around *T* = 160°C, a phase transition occurred during the deposition process which kept the sputtered Bi in the liquid state for a sufficient amount of time. During this time, the stronger cohesion of the liquid Bi than the adhesion to the glass surface started to give these nanoparticles the ability to clear the neighborhood around them. The cohesion of the liquid Bi becomes higher with temperature. This gives the explanation to the fact that while the sample grown at 160°C (Bi-104) has BiNPs with apparent edges and corners, the sample grown at 200°C (Bi-105) has BiNPs with spherical shape. Although samples grown over 200°C (Bi-106) did show BiNPs, the results were unstable as the temperature approached the melting point of Bi (271.4°C). The maximum possible temperature to grow a BiNP sample is 250°C, with most Bi composites vaporized after this point. The above results show that the best substrate temperature for feasibly making size-controllable BiNPs is 200°C, which leads us to the next stage of our experiment.

**Figure 2:**
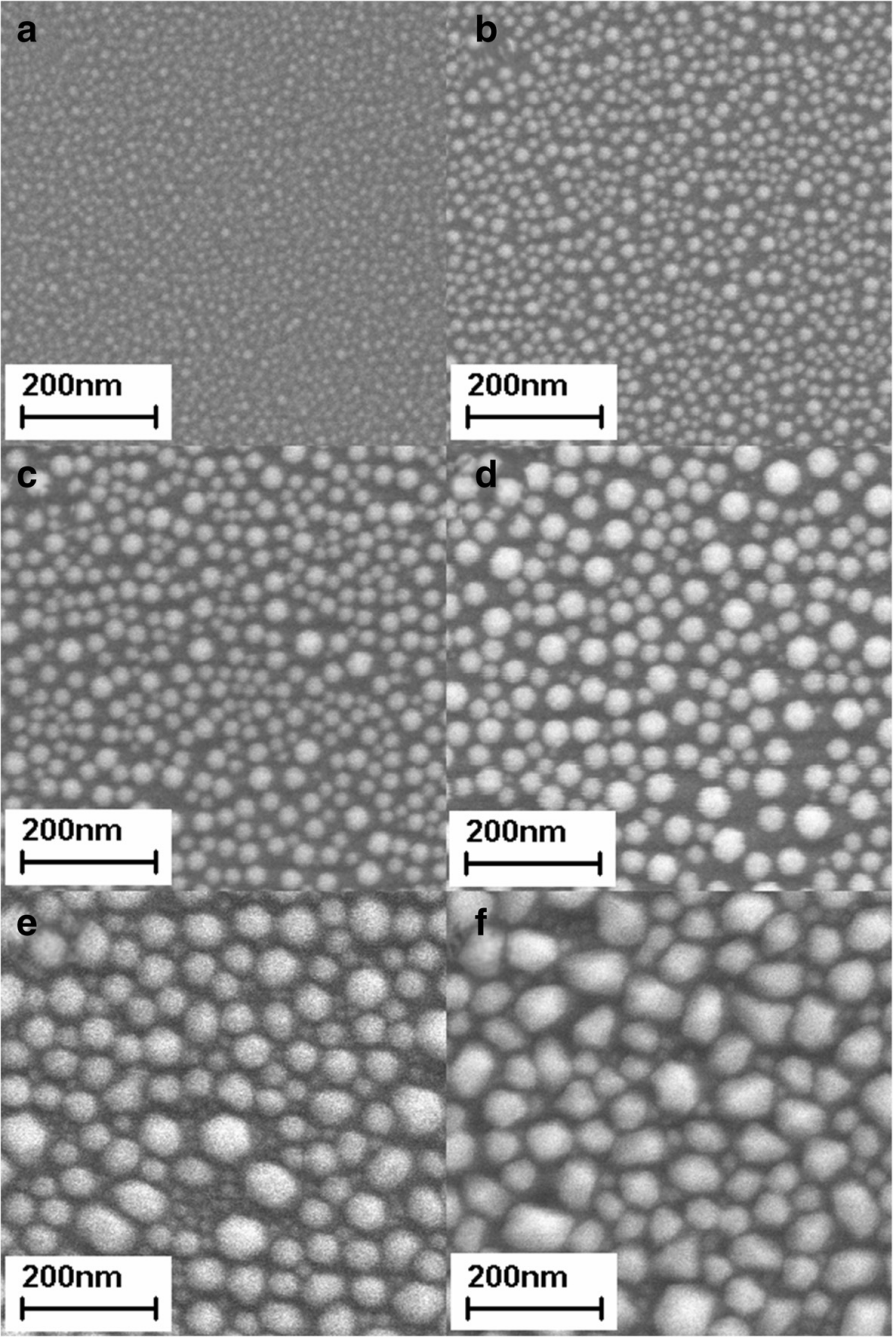
**SEM images of BiNPs deposited on glass substrates at 200°C and 0.12 W/cm**^**2 **^**for different deposition durations. (a)** 10 s, **(b)** 20 s, **(c)** 30 s, **(d)** 40 s, **(e)** 50 s, and **(f)** 60 s, which correspond to sample Bi-201 to Bi-206 in Table 1.

SEM images of BiNPs grown at 200°C and 0.12 W/cm^2^ for different deposition durations (10 to 60 s) are shown in Figure 2a,b,c,d,e,f. Unlike the thin film-like samples grown at low temperatures, all samples grown at 200°C showed distinct particle-like BiNPs. By depositing samples at this temperature with different durations, we were able to control the size of the BiNPs. Furthermore, samples deposited for shorter durations (10 to 40 s) showed spherical-shape BiNPs, but samples deposited for longer durations (50 and 60 s) showed crystal-like BiNPs. This crystallization behavior can be identified by the XRD pattern (figure not shown here). The ratio of the diffraction peak of the preferred orientation to the other minor peaks becomes stronger as the deposition duration increases.

**Figure 3:**
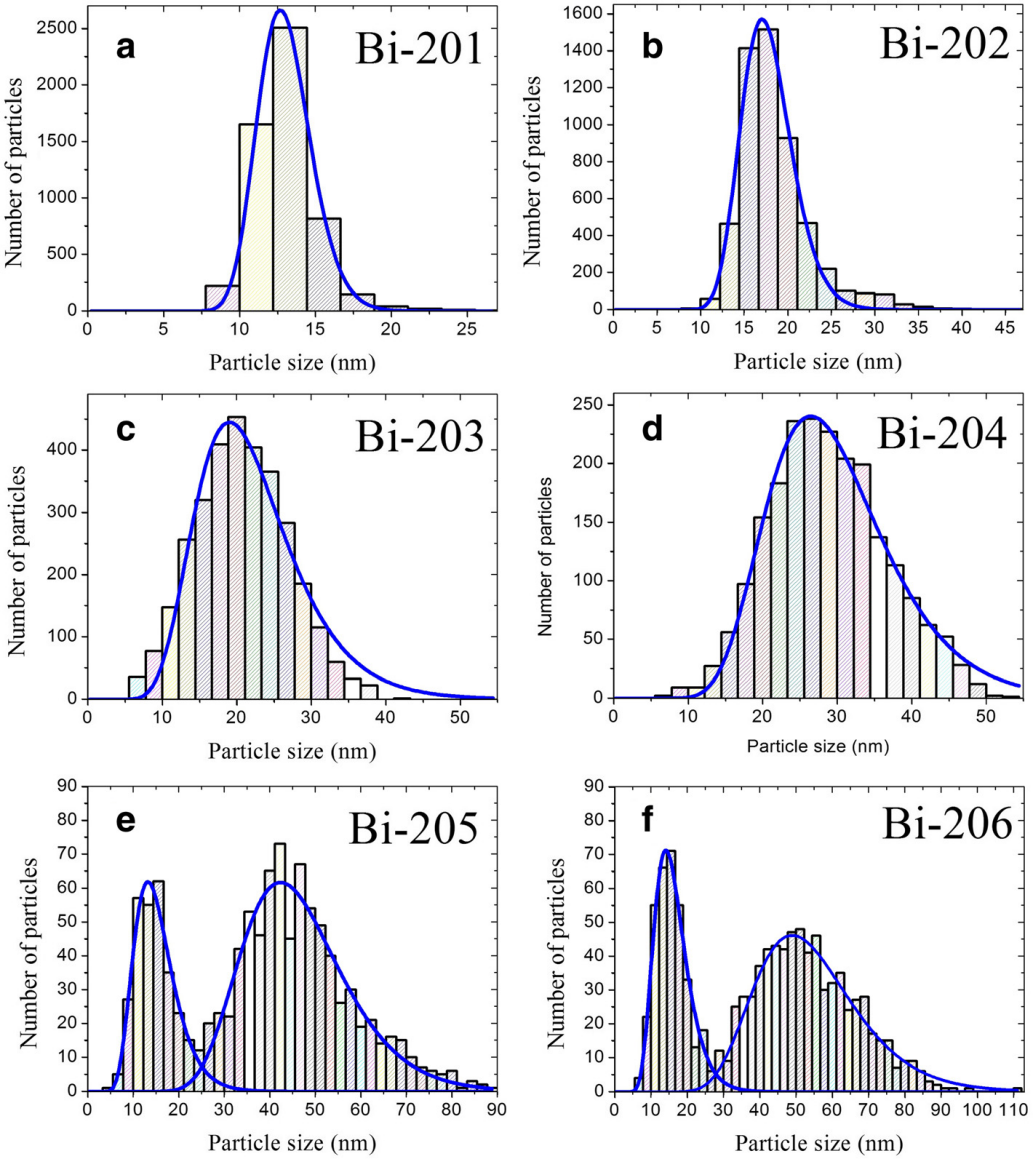
**Particle size distribution statistics and lognormal fitting. (a**-**d)** Samples Bi-201 to Bi-204, and **(e**, **f)** samples Bi-205 and Bi-206. The analysis is carried out from SEM images within a fixed area of 3.5 μm^2^.

Particle size distribution of samples Bi-201 to Bi-206 can be obtained by measuring the diameters from the SEM images. We use a simple computer program to examine every SEM image file pixel-by-pixel, and the shapes of the BiNPs are identified by their color differences (the color on the substrate is darker than on the nanoparticles). By summing up the pixels, the area of each nanoparticle can be determined, and thus its diameter. In this way, we can calculate the mean diameter of any form within a fixed area of 3.5 μm^2^. The results are shown in Figure 3, and some of the important statistical values are listed in Table 3. Note that the average diameter of the sample is $\bar{d}$*,* the standard deviation of each sample is $\bar{d_{v}}$, and the peak of lognormal fitting [27] is $\bar{\mathrm{d\mu}}$, which corresponds to the mode. There are apparently two lognormal fitting peaks for 50- and 60-s deposited samples, which means that there exist two particle sizes. During the sputtering process, two BiNPs merged to form a larger particle, so extra space emerged as new BiNPs begin to grow. Minimum area for BiNP nucleation can therefore be estimated to be 2.5 × 10^2^ nm^2^. As a consequence, all of the abovementioned statistical parameters ($\bar{d}$, $\bar{d_{v}}$, and $\bar{\mathrm{d\mu}}$) increase with the deposition time, but the time dependence of the BiNP density shows a minimum density at 40 s.

**Table 3:** Particle size statistics and estimated bandgap of samples Bi-201 to Bi-206

**Number**	$\bar{\mathrm{d\mu}}$**(nm)**	$\bar{d}$**(nm)**	$\bar{d_{v}}$**(nm)**	***E***_***g ***_**(eV)**
Bi-201	12.9	13.0	13.2	2.63
Bi-202	17.5	18.5	18.9	2.50
Bi-203	21	20.7	21.7	0.85
Bi-204	28.7	28.9	29.9	0.91
Bi-205	14.5 and 45.1	38.3	42.3	1.39
Bi-206	15.3 and 52.6	41.1	45.9	0.45
Bi-101				0.18

The bandgap of sample Bi-101 (Bi thin film) is listed as a reference.

The optical bandgap of each sample can be estimated by using the Tauc equation [28]:

(1) $${\left(\mathrm{ahv}\right)}^{n}=A\left(\mathrm{hv}-E_{g}\right)$$

where *a* is the absorption coefficient, *hv* is the photon energy, the exponent *n* depends on the nature of the transition (in our case, *n* = 1/2 corresponds to the indirect bandgap material [29]), *A* is a constant, and *E*_*g*_ is the optical bandgap. Traditionally, in thin film samples, *a* is determined by the equation of transmission *T* = *e*^−*ad*^ if we neglect the surface and internal multiple reflections, where *T* is the transmission coefficient and *d* is the thickness of the film. The Tauc equation is usually used to measure the bandgaps of thin film samples. However, as long as the density of the nanoparticles is high enough, this method is also a good approximation to estimate the bandgaps of nanoparticle samples [30,31]. For a more precise estimation, we adopt another method to calculate α for these samples. Consider light passing through a sphere with radius *r* in the spherical coordinate system (*θ* being the polar angle). The vertical distance for the light to travel through the sphere is *d* = 2*r*cos*θ*, and the projected shadow area of the angle *dθ* is *dA* = 2*πr*^2^cos*θ*sin*θdθ*. With *I*_0_ being the intensity per unit area, the differential intensity of this area *dI* can be described as

(2) $$\mathrm{dI}=I_{0}\mathrm{dA}=I_{0}e^{-2\mathrm{\alpha r}\cos\theta }2\pi r^{2}\cos\;\theta \sin\;\mathrm{\theta d}\;\theta$$

By deciding *T*, we can calculate *a* by the following equations:

(3) $$TI_{0}\pi r^{2}=\int_{0}^{\frac{\pi }{2}}I_{0}e^{-2\mathrm{ar}\cos\theta }2\pi r^{2}\cos\;\theta \sin\;\mathrm{\theta d}\;\theta$$

(4) $$T=-\frac{1}{\mathrm{\alpha r}}e^{-2\mathrm{\alpha r}}+\left(\frac{1}{2\alpha^{2}r^{2}}-\frac{1}{2\alpha^{2}r^{2}}e^{-2\mathrm{\alpha r}}\right)$$

**Figure 4:**
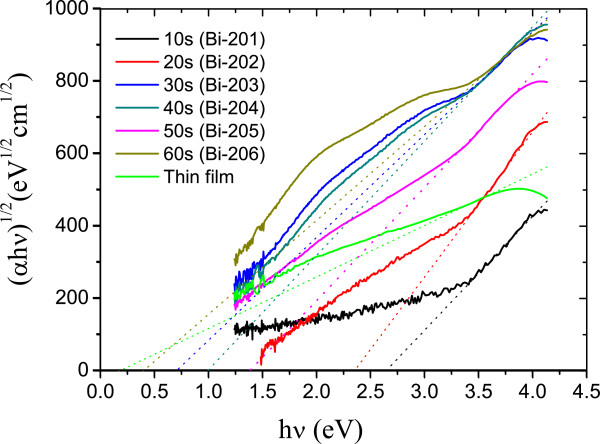
**Plot of (*ahv*)**^**1/2 **^**vs. (*hv*)for the estimation of indirect bandgap of Bi-201 to Bi-206 and Bi-101.** The absorption coefficient α is calculated through the optical transmission spectrum. Dashed lines indicate the extrapolation of the data for optical bandgaps. The inset shows the schematic diagram of light passing through a nanoparticle.

We measured the optical transmission spectrum of samples with BiNPs (Bi-201 ~ Bi-206) and Bi thin film (Bi-101) ranging from 300 to 1,000 nm. These data are presented by using a Corning glass as a reference. At higher wavelength, *T* decreases as the deposition time increases. The absorption edges also shift toward a longer wavelength, indicating a possible bandgap modulation by controlling the size of BiNPs. Figure 4 shows the plot of (*αhν*)^1/2^ vs. (*hν*), and the estimated bandgaps are determined by the extrapolation (dashed lines) through these curves. The values are listed in Table 3. The bandgap decreases as the diameter of BiNPs increases. The results are reasonable compared with the data acquired by Selzer's group [32], in which the bandgap of 3-nm BiNPs was measured by other methods to be approximately 2 eV.

**Figure 5:**
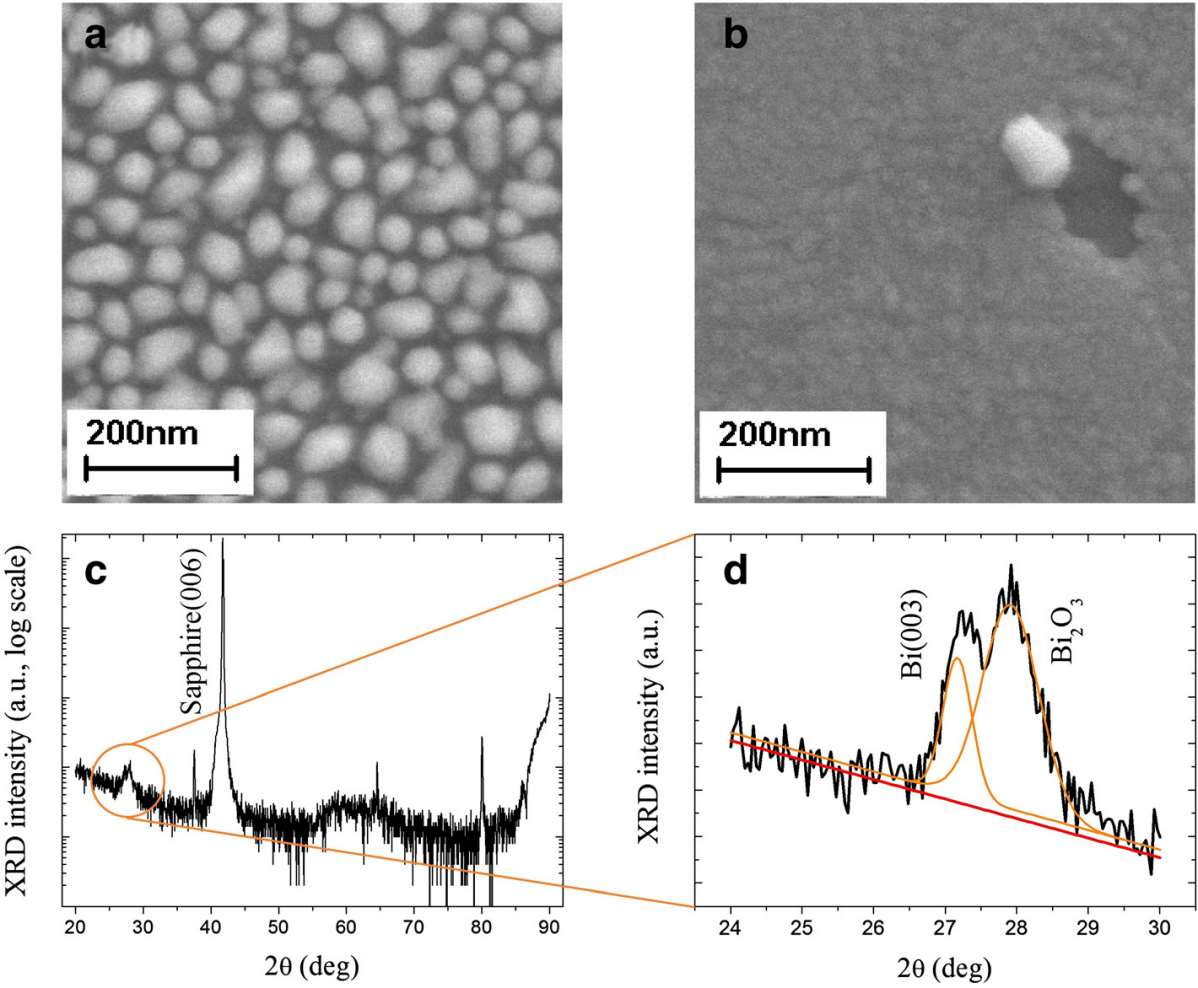
**SEM images and XRD spectra in experiment C. (a)** SEM images of BiNPs deposited on ITO glass substrates at 160 °C (Bi-301). **(b)** SEM images of BiNPs deposited on ITO glass substrates at 200°C (Bi-302). **(c)** XRD spectra of the BiNPs prepared on c-plane sapphire at 200°C and 0.12 W/cm^2^ for 60 s (Bi-304). **(d)** A closer look from 2*θ* = 24*°* to 2*θ* = 30*°*, in which Bi(003) and Bi_2_O_3_ diffraction peaks can be identified.

Through chemical reactions with substrates, the quality of BiNPs can be different. The third and final stage of our experiment was to deposit Bi on different substrates (ITO glass and c-plane sapphire). The SEM images of the Bi deposited on ITO glass and on sapphire at low temperatures (below 200°C) show BiNPs of more crystal-like shape, with a density higher than the ones deposited on glass substrates. However, at 200°C, 0.12 W/cm^2^, and 60 s, only Bi thin films were deposited. This can be seen in Figure 5a (Bi-301) and 5b (Bi-302). The reduction of the formation of BiNPs is due to the oxidation with the substrates. High-resolution XRD spectrum of the BiNPs prepared on c-plane sapphire at 200°C (Bi-304) is shown in Figure 5c. A sharp peak can be ascribed to Al_2_O_3_ (006) at 2*θ* = 42°, together with a broadened minor peak at 2*θ* = 27.5°. A closer look from 2*θ* = 24° to 2*θ* = 30*°* shown in Figure 5d reveals that this minor peak can be considered as the combination of two distinct peaks, Bi (003) at 27.17° and Bi_2_O_3_ at 27.92°. The same conditions occurred on BiNPs deposited on ITO glass. Since pure bismuth samples suffer oxidation gradually, as can be checked by the XRD spectrum measured day by day, we can thus rule out the possibility that the samples were oxidized after they were taken outside. This oxidation effect can be explained by comparing the bonding energies of oxygen with other elements [33-35]. The bonding enthalpies (in unit of kJ/mol) are 337.2 ± 12.6 for Bi-O, 320.1 ± 41.8 for In-O, 531.8 ± 12.6 for Sn-O, 511 ± 3 for Al-O, and 799.6 ± 13.4 for Si-O. As can be clearly seen from this table, the bonding enthalpy between Bi and O is significantly lower than the values between O and other elements, except In-O. This indicates that Bi_2_O_3_ can be formed easier than SiO_2_, Al_2_O_3_, In_2_O_3_, and SnO_2_. Once the temperature during deposition process is high enough, the bonding between Al-O, In-O, and Sn-O may be weakened and increase the possibility of the formation of Bi_2_O_3_. On the other hand, Si-O bonding is too high for the oxidation process to take place. We thus conclude that once the substrate temperature is high enough, Bi can react with oxygen from substrates to form Bi_2_O_3_, which compromises its ability to form BiNPs.

## Conclusions

We present a systematic experiment to measure the optimal conditions to grow a single layer of BiNPs on various substrates by using a RF sputtering system at 200 °C, using 0.12 W/cm^2^. With suitable chosen parameters, BiNP samples were successfully fabricated, instead of BiNWs and Bi thin films. Since the optical bandgap decreases as the diameter of BiNPs increases, we were able to modulate their values by depositing various sizes of BiNPs. All these data and sample statistics are listed in the tables for future references.

## Abbreviations

BiNPs: bismuth nanoparticles; BiNWs: bismuth nanowires; ITO: indium tin oxide; RF: radio frequency; SEM: scanning electron microscope; XRD: X-ray diffraction.

## Competing interests

The authors declare that they have no competing interests.

## Authors' contributions

HYL and BKW conceived the study and drafted the manuscript. MYC coordinated the projects. BKW helped with the preparation of Bi nanoparticles. BKW and HYL helped with the FESEM, XRD, and optical transmission spectra characterization. All other works were carried out by BKW. All authors read and approved the final version of the manuscript.

## Authors' information

HYL obtained his Ph.D. degree at National Taiwan University (NTU) and is currently a post-doctoral fellow of the Department of Physics, NTU. BKW obtained his Ph.D. degree at NTU and is currently a post-doctoral fellow of the Institute of Biomedical Engineering, NTU. MYC obtained his Ph.D. degree at Cornell University, USA, and is currently a professor of Physics, NTU.
